# Supplementary material for: Facilitated Photocatalytic Degradation of Rhodamine B over One-Step Synthesized Honeycomb-Like BiFeO3/g-C3N4 Catalyst
Source: Nanomaterials (Basel). 2022 Nov 10;12(22):3970. doi: 10.3390/nano12223970 (PMC9699307; doi:10.3390/nano12223970)
Supplement: Supplementary file 1 [file nanomaterials-12-03970-s001.zip › nanomaterials-1969941-supplementary.pdf]

Supplementary Materials

Facilitated Photocatalytic Degradation of Rhodamine B over One-Step Synthesized Honeycomb-like $BiFeO_3/g\text{-}C_3N_4$ Catalyst

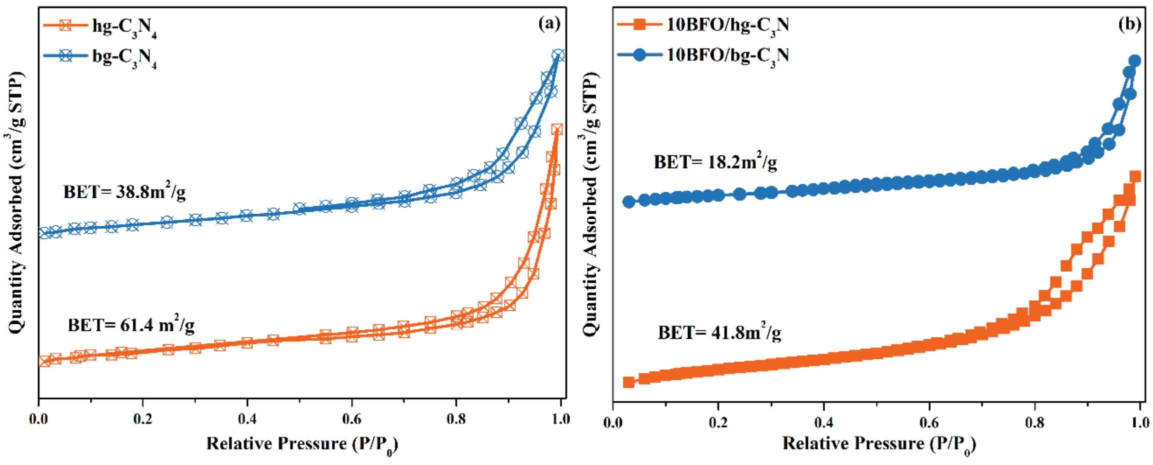

Figure S1. N_2 physisorption isotherms of (**a**) bg-C_3N_4 and hg-C_3N_4; (**b**) 10BFO/bg-C_3N_4 and 10BFO/hg-C_3N_4.

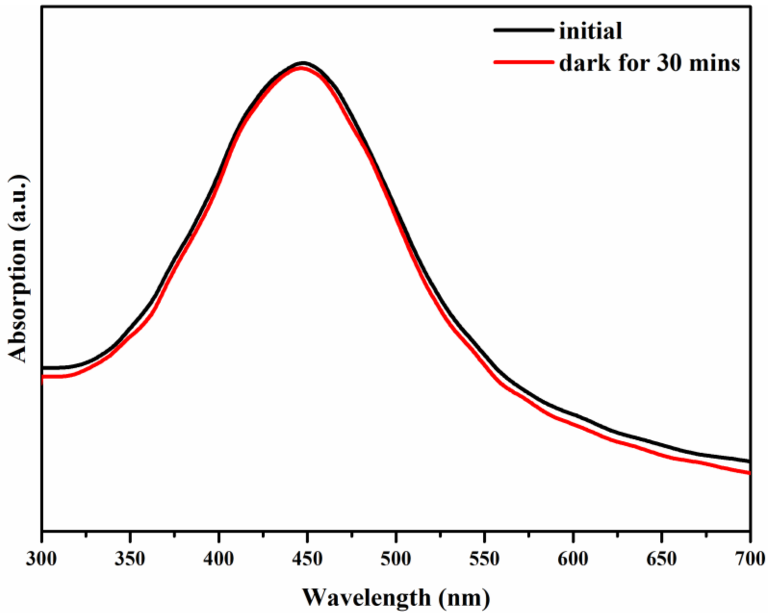

Figure S2. UV-Vis spectra of initial methyl red solution and after dark adsorption-desorption equilibrium for 30 minutes with 10BFO/hg-C_3N_4.

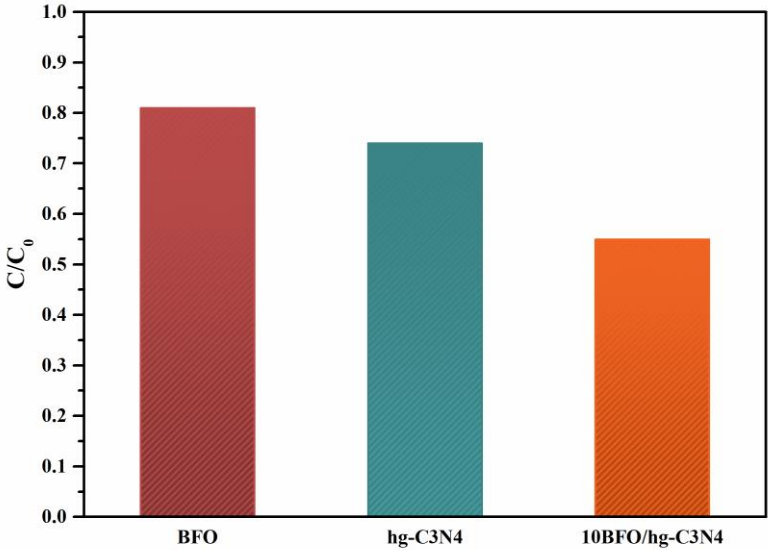

Figure S3. Photodegradation profiles of methyl red with different catalysts under solar simulator within 60 min.

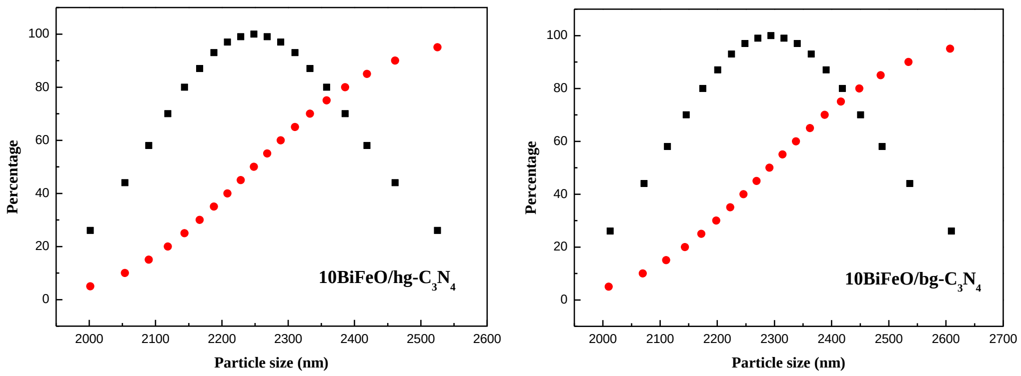

Figure S4. The particle size distribution of the samples.

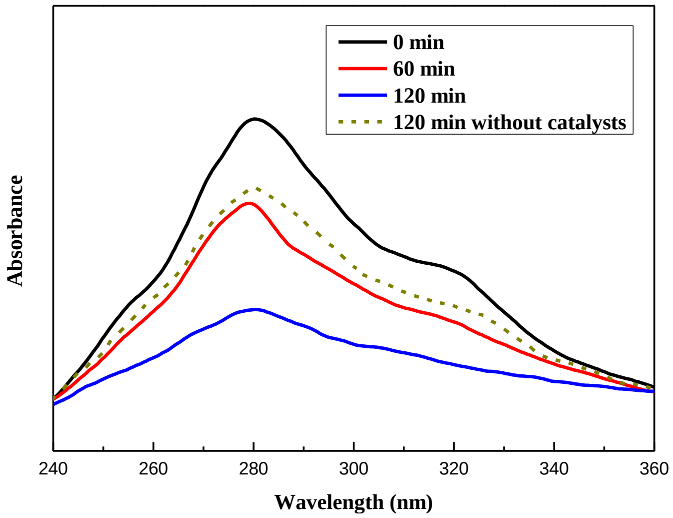

Figure S5. Disappearance of coumarin under irradiation.
